# Centargo Equipped with Smart Protocols Versus Stellant Injectors: A Comparison of the Impact on Contrast Media Usage, Sustainability, and Workflow Efficiency in CT Scan Suite

**DOI:** 10.1007/s10278-025-01563-x

**Published:** 2025-06-04

**Authors:** Yohan Anquetil, Thibaut Leturgez, Jerome Jacquin, Francois Kruta, François Jambon

**Affiliations:** 1 CIMROD, Perigueux, France; 2 Bayer, France; 3 Ubudu, France

**Keywords:** Centargo, Contrast media, CT injectors, Radiology, Personalized protocols, Weight-based dosing, Low KV imaging, Workflow efficiency, Environmental sustainability, Patient care

## Abstract

This study evaluates the impact of Multi-use practices in computed tomography (CT) and MEDRAD® Centargo injectors equipped with Smart Protocols and other functionalities aimed for optimizing contrast media consumption, workflow efficiency, patient care time, and environmental sustainability at the CIMROD center. Data were collected across three measurement periods, assessing contrast volume per patient, distribution of activity on several tasks, injector preparation time, time for Iodinated contrast media documentation for traceability, and waste measurements. The Centargo injector equipped with Smart Protocols and kV optimization reduced contrast volume from a target of 80 mL to an average of 72.98 mL using Smart Protocols, then further optimized to 69.95 mL using kV optimization. Compared to previous injector generation, there was a decrease in non-added value tasks such as injector preparation time by 47.3%, measured with Udubu, or 72.35 s, measured by chronometer, per injected procedure. The multi-use implementation reduced waste production by 69% and 74%, respectively, for Centargo and Stellant multi-use versus single-use injectors. Even if the study has limitations, injectors such as Centargo equipped with Smart Protocols and other functionalities enhance operational and financial efficiency along with sustainability in radiology and contributes to clinical practice evolution.

## Introduction

The radiology landscape in France is undergoing a significant transformation, particularly regarding the supply model for contrast media enhancement products used in medical imaging [1]. With the March 1, 2024, transition, there was an urgent need to reevaluate the functionalities and efficiencies of CT injectors, which are essential for optimizing imaging procedures by managing ICM consumption in accordance with the “As Low As Reasonably Achievable” (ALARA) principles [2] to enhance patient safety, indirectly reduce patient and Radiographers’ exposition to radiation while maintaining diagnostic accuracy and also the opportunity to improve sustainability, financial performance, and the suite workflow.

A deeper understanding of ICM pharmacokinetics, as highlighted by Bae et al. in 2010 [3], emphasizes the importance of weight-based dosing and personalized imaging protocols. Research, including a 2024 study by Thuering et al. [4], shows that tailored protocols can reduce ICM dosage by 19.2% compared to fixed-dose methods. Additionally, low kilovolt (kV) scanning techniques decrease radiation doses and allow for further reductions in ICM dosing due to increased attenuation. The French Society of Radiology’s 2020 CIRTACI (Comité Interdisciplinaire de Recherche et de Travail sur les Agents de Contraste en Imagerie) recommendations based on several key publications [5–9] state that a 20 kV reduction can theoretically decrease both ICM and radiation doses by about 20%.

The implementation of multi-use vials and injectors’ consumables, for Stellant and Centargo, minimizes the unused or overused ICM, which is not measured in this study, while also reducing the number of vials utilized and their environmental impact. From a sustainability perspective, using an injector designed for multi-use allows for less consumables. A daily kit is used with patient lines that are replaced for each patient compared to syringes and patient lines for every patient.

The Centargo injector represents a new generation of technology tailored to meet the evolving demands of radiology departments. It offers advanced features aimed at improving operational efficiency, reducing operational costs, and enhancing patient care outcomes. By automating tasks such as purging, injection preparation, and worklist connectivity between acquisitions, the Centargo injector alleviates activity pressure on radiographers. Additionally, it enhances injector controls for patient safety such as pro-active air management, which in turn helps to reduce potential anxiety among radiographers.

The Radiology Information System (RIS)-injector connectivity automates, and so, avoid Radiographer to spend time on ICM documentation for traceability purpose that, while necessary, do not add direct clinical value to radiographers’ work. The RIS is essential for managing daily operations in medical imaging, including patient scheduling, exam report storage, result distribution, and billing. Radiographers must document the volume of contrast media injected, along with the brand and batch number, in the RIS for traceability, which is crucial for identifying defective batches during patient incidents. However, manual traceability can lead to human error and is time-consuming under clinical pressure. Automating these processes with the RIS-injector connection improves accuracy and saves valuable time for the radiographer team.

Given the expectations for personalized ICM consumption supported by Smart Protocols software, the workflow improvements facilitated by the Centargo injector through integrated task automation and RIS-injector connectivity, the remote access to injector activity data with the Virtual-Care® Nautilus software, the sustainability benefits from reduced consumable waste, and the potential lower electricity consumption linked to kV optimization, the Centre d’Imagerie Médicale de la Dordogne (CIMROD) has recently integrated two Centargo injectors into its operations. This integration provides an ideal setting for evaluating these advancements and launch the study which have as objectives to measure how the equipment, as the whole, impacts the ICM consumption, the radiographers’ time and the production of waste. This study is offering valuable insights that will inform the suite practice, enhance decision-making, and support potential cost-effectiveness analyses in the context of evolving healthcare policies in France and beyond.

## Materials and Methods

The comprehensive implementation of the CT scan solution at the CIMROD center included MEDRAD® Centargo Injectors, Ultravist 370® (iopromide; Bayer AG, Germany) as the ICM, Smart Protocols® software, Virtual-Care® Nautilus software, Application Specialist Intervention, and a two-way RIS-Injector connection. The equipment as a whole was expected to optimize of ICM consumption, enhance operational workflows, and allow radiographers spending more time with patient.

Data for this study was meticulously collected over a 5-month period (from April 25, 2024, to September 13, 2024). During that period, 5 sources of data were collected marking the transition from the Stellant single-use injector to the Stellant multi-use and finally Centargo injector equipped with multiple software. Over the 5 months, the Radiographer team, the patient recruitment, the Radiologist team, and the injector location in the suite remained the same.

ICM volume via Nautilus and radiographers’ positions collected via the Ubudu®’s Real Time Location System (RTLS) were continuous and remotely collected. The Ubudu tagging system provides precise indoor localization solutions, generating position in the CIMROD suite every second. Each radiographer wore an anonymized tag, allowing for accurate location tracking within one meter during their shift. Ubudu’s tags generated positions when they were unplugged from charging, allowing for continuous tracking and limiting overall noise.

On top on continuous and remote data collection, three on-site measurements corresponding to the 3 injectors set up system were done: April 25 and 26 for Stellant single use, May 6 and 7 for the Stellant multi-use, and July 10 and 11 for Centargo. During the on-site interventions, the time required for injector preparation, the time dedicated to ICM documentation for traceability at each examination, and finally the waste weight generated were manually collected by independent observer. The independent observer could collect data for 1 injector at the time. In the meantime, radiographers’ activities were tracked by the Ubudu system excepted for the Stellant single use measurement which was not operational at that time.

The primary endpoint was to focus on injection volume administered by radiographers with the support of injectors. According to the injector data collection system Nautilus, those data are anonymized and aggregated. As a result, patients’ consent was not required. Autodoc raw data enabled retrieval of the study bundle or injection level and anonymized data. The data collected from the injectors and sent to the Nautilus Cloud are hosted in Europe and comply with GDPR prerogatives.

After Centargo’s installation, Bayer’s Application Specialists performed two interventions including one in collaboration with the Original Equipment Manufacturer (OEM) engineer to optimize imaging protocols. The interventions were organized by the CIMROD Radiology team, including a Radiologist for image quality control and a Radiographer for protocol standardization. Pre-defined protocols were implemented in Smart Protocols software. It consisted of pre-configuring protocols including weight-based and kV rules adapting the dose. The CT scanner kV and milliampere settings were also optimized to the new pre-defined protocols and in accordance with CIRTACI’s recommendations. The tri-parties collaboration ensured that dose reductions did not negatively impact ICM enhancement on hepatic parenchyma’s ROI and, ultimately, image quality, an essential outcome for diagnostic purposes.

The number of expected injections per day was calculated according to Nautilus data used in the ICM consumption analysis (Table 1). During period 1, from June 3, 2024, to August 6, 2024, it represents practices after the implementation of Centargo with Smart Protocols and the intervention of Bayer’s application specialist. This period represents 65 days and 47 working days during which 1337 injections were done leading to 28.44 injections per day. During period 2, from August 7, 2024, to September 13, it reflects the practices after the implementation of Centargo, Smart Protocols, and kV associated with Milliampere adjustment by the co-intervention of Bayer’s application specialist and the OEM engineer. This period represents 37 days and 27 working days (15 th of August was a bank holiday) during which 768 injections were done leading to 28.44 injections per day. So, for other analyses of this study, a mean number of 28 injections per day was selected. No longitudinal data regarding ICM consumption was collected with either of the Stellant injectors.

**Table 1:**
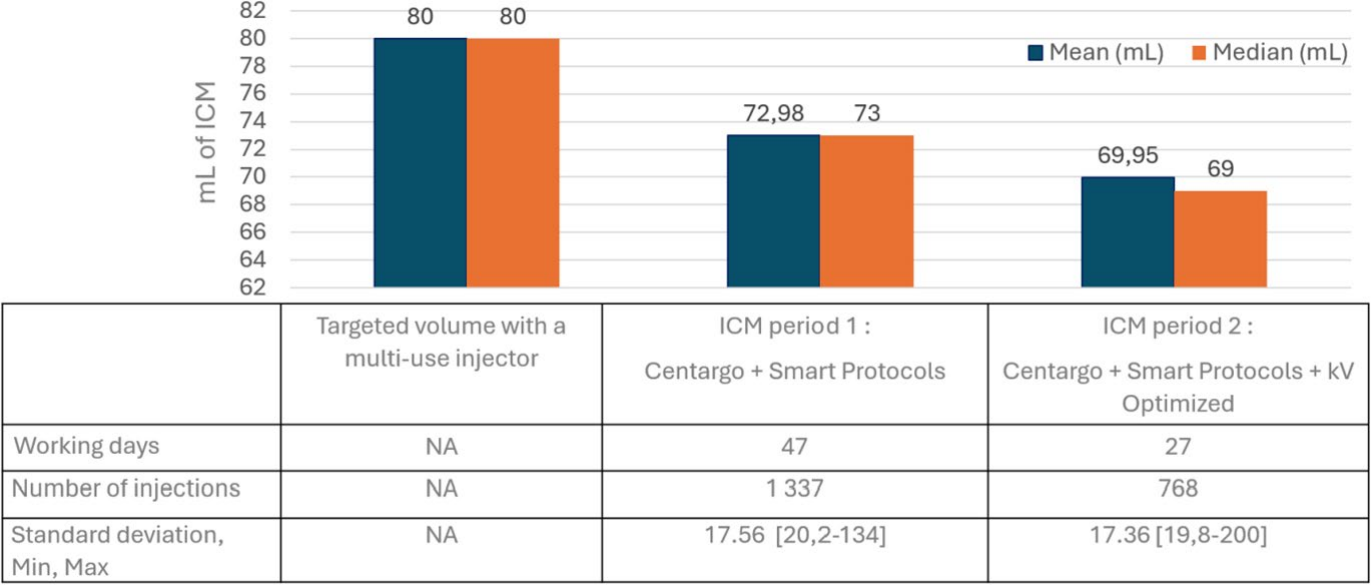
Results of the ICM consumption in mL, comparison of the Centargo and Smart Protocols period to the Centargo and Smart Protocols, and kV optimization versus the initial targeted ICM volume

Regarding secondary endpoints and to demonstrate Radiographers’ activity distribution on each task, Ubudu tagging system was used. The aim was to demonstrate a reduced activity spent on non–added-value tasks (such as injector preparation and ICM documentation for traceability) and reallocated effort toward added-value activities such as post-treatment processing and probably direct patient care. Two injector configurations through two periods were compared. The Stellant multi-use period from May 6, 2024, to May 26, 2024, is representing 12 working days (on top of weekend, 8 and 20 of May were bank holidays in France) against the Centargo injector period from June 3, 2024, to September 13, 2024, is representing 72 working days (15 of august was a bank holiday). A transition period during which Stellant and Centargo co-exist was excluded from the analysis, from May 27, 2024, to June 2, 2024.

**Fig. 1:**
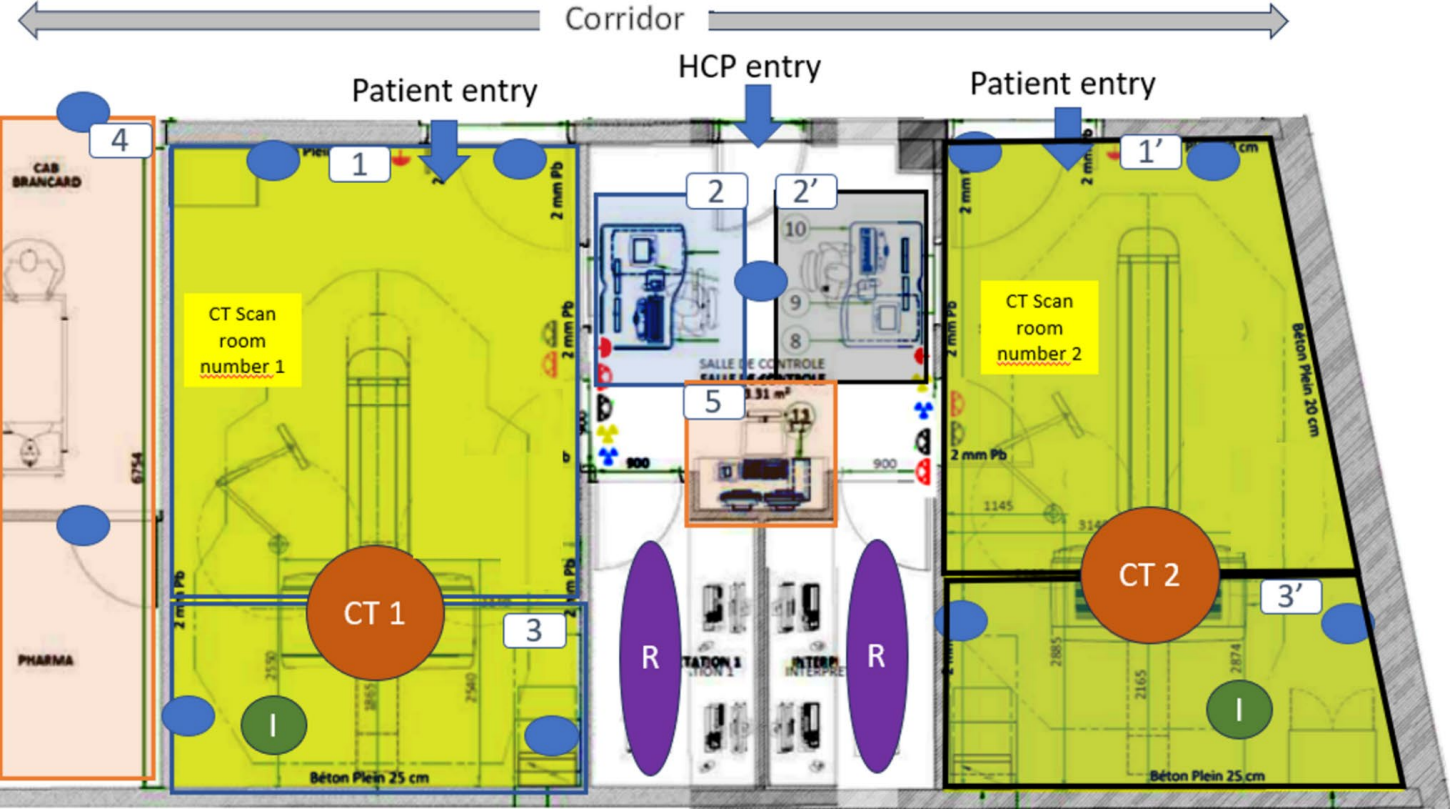
CIMROD CT scan suite map with the Ubudu setup. (CT1 = CT scanner number 1, CT2 = CT scanner number 2, I = injector, R = radiologist interpretation zones, 1 and 1′ = patient management zones, 2 and 2′ = control desk zones, 3 and 3′ = injector preparation zones, 4 = pharmacy zone, 5 = RIS management zone (used for the billing and traceability), blue dot = Ubudu anchor placements)

For the effective use of Ubudu’s tagging system, the CIMROD CT scan suite map (Fig. 1) was essential. It outlines the activities of radiographers associated with specific area markings. Five zones of interest were established, representing key tasks in their daily routines. We excluded the cannulation activity due to its limited relevance to the injector’s impact on workflow and the inclusion of zones used by radiographers for breaks. CIMROD operates two CT scanners, and some identified zones are shared between the two scanner rooms. On the map, zones are indicated with a “′” to denote their association with CT Scan room 1 or room 2 (for example, 2′ indicates the control desk zone for CT Scan room 2).

Tags’ positions on the map which are generated on a second-based were collected over specific periods, calculated in average position per day, and categorized by percentage per zone. During a typical shift at the CIMROD site, three radiographers collaborated closely. They wore tags throughout standard daily shifts from 8:30 a.m. to 5:30 p.m. with a 1-h launch break, excluding night and weekend shifts. Positions recorded during breaks and patient cannulation outside of the designated zones were excluded from the analysis.

Data recovery from the Ubudu system was conducted using a cloud-based solution, facilitating efficient data management and utilization. Radiographers participating in this study signed consent forms, and their data were aggregated to ensure confidentiality and respecting GDPR.

The time radiographers spent on injection preparation and ICM documentation for traceability was manually measured by an external observer between different injector setups and by using a chronometer. For Stellant multi-use and Centargo, those measurements occurred concurrently with the Ubudu’s measurement and were initially thought to back Ubudu’s data. Injector preparation time included all tasks related to preparing the injector, such as spiking the vial, preparing consumables, filling the injector with ICM, adjusting the injection protocol based on patient weight, and connecting the patient line to both the injector and the patient’s catheter. The traceability activity was conducted on a computer dedicated to RIS management and also billing activities. The chronometer was started only when the radiographer accessed the computer to perform ICM documentation for traceability.

The independent observer attended April 25 and the morning of the 26, so a maximum of 42 (28 × 1.5) data point would have been collected. For the May 6 and the morning of the 7, so a maximum of 42 data points would have been collected. For the July 10 and 11, a maximum of 56 (28 × 2) data points would have been collected. The actual number of injections and data point collected in Table 3 is lower to the maximum achievable data point collection. Those numbers were dependent to the rate of injected procedures, difficulties collecting accurate data due to the random occurrence of injected procedure, intensity of the activity, and no-shows.

The waste generated from different injectors setups was quantified by weighing empty products, including vials and consumables, after use with a household scale for comparison across different injector setups. Those data were generated during 3 on-site intervention, April 25 and 26 for Stellant single use, May 6 and 7 for the Stellant multi-use, and July 10 and 11 for Centargo. Data allowed for extrapolation of theoretical waste production per day and per year for each injector.

## Results

At CIMROD, the target contrast volume was set at 80 mL using Ultravist 370. To assess the statistical significance, one-sided t-tests (*α* = 0.025) were conducted under the null hypothesis that the mean injection volume is ≥ 80 mL, against the alternative hypothesis that it is < 80 mL.

During ICM period 1, the Centargo injector achieved an average volume of 72.98 mL (median: 73.0 mL; standard deviation: 17.56 mL; range: 20.2–134.0 mL) per injection after 1337 injections, resulting in a mean reduction of 8.77% compared to the 80 mL baseline. During ICM period 2, this average volume decreased to 69.95 mL (median: 69.0 mL; standard deviation: 17.36 mL; range: 19.8–200 mL), reflecting a significant 12.56% reduction compared to the initial target. The results for both protocols were statistically significant (*p* < 0.0001) (initial target vs. period 1 and period 1 vs. period 2), indicating that the mean injection volumes were significantly lower than the 80 mL threshold. (See Table 1 for detailed results.) Note that Smart Protocols is the main driver to optimize ICM volume.

The Wilcoxon test was used for statistical testing, two-sided with an alpha of 0.05. The data is not normally distributed, and the total number of positions per day on the map is decreasing by approximately one third between the 12 working days of Stellant multi-use and the 72 working days of Centargo (43,381 vs. 29,049). The *t*-test was used on the averages, and the numbers were taken from the daily measurements. Due to the exploratory testing of the Ubudu system and those differences, statistical results need to be interpreted cautiously. The numerical evidence suggests that Centargo, with its integrated Smart Protocols and RIS-injector connectivity, is effective at reducing the number of daily positions in zones dominated by non–added-value tasks. This reduction is particularly prominent in areas such as injector preparation, pharmacy, and RIS and ICM documentation for traceability.

Due to that limitation, the percentage of positions per zone seemed to be more reliable for interpretation. As results were measured, a 47.3% reduction in positions related to injector preparation, 27.5% of positions related to the RIS and ICM documentation for traceability zone, an 11.4% increase in position in the zone where radiographers are taking care patient, and a 4.4% increase of positions at the control desk which was associated by the Radiographer’s team to activity was dedicated for post image acquisition management and preparing radiologist interpretation leading to efficiency improvement (see Table 2 for detailed results).

**Table 2:** Result of the Ubudu tagging system

	Ubudu period: Stellant multi-use (12 days)		Ubudu period 2: Centargo (72 days)		Comparison of periods		
Zone name	Average number of positions on the map per day	Percent	Average number of positions on the map per day	Percent	*p*-value on average position per day	The net change in percent	Evolution in percent
Pharmacy	2776	6.4%	1959	6.5%	**0.030**	**0.1%**	** ~ 0%**
Traceability/ICM documentation + RIS management	1749	4%	829	2.9%	** < 0.0001**	** − 1.1%**	** − 27.5%**
Control desk (CT scan rooms 1 and 2)	16,648	38.4%	11,603	40.1%	** < 0.0001**	** + 1.7%**	** + 4.4%**
Injector preparation (CT scan room 1 and 2)	4776	11%	1680	5.8%	** < 0.0001**	** − 5.2%**	** − 47.3%**
Patient care (CT scan rooms 1 and 2)	17,431	40.2%	12,978	44.8%	** < 0.0001**	** + 4.6%**	** + 11.4%**
Total	43,381	100%	29,049	100%	**NA**	**NA**	**NA**

The statistical test results on average position per day and the percentage calculation results consistently indicate that the task distribution of radiographers shifts favorably with Centargo compared to Stellant. There is a clear reduction in non–added-value activities (such as injector preparation and ICM documentation for traceability) to added-value activities (such as patient care and control desk) (Table 3).

**Table 3:** Result of chronometer time allocated for the injector preparation and ICM documentation for traceability

	Injector preparation				RIS and traceability			
Injector type	No. of records	Mean	Median	SD, Min, and Max	No. of records	Mean	Median	SD, Min, and Max
Stellant single-use	33	1 min 32 s	1 min 29 s	0 min 25 s [30 s–3 min 12 s]	31	14 s	13 s	5 s [7 s–30 s]
Stellant multi-use	24	1 min 39 s	1 min 16 s	1 min 6 s [32 s–4 min 23 s]	23	23 s	19 s	14 s [9 s–1 min 2 s]
Stellant single + multi-use	57	1 min 35 s	1 min 28 s	47 s [30 s–4 min 23 s]	54	17.6 s	14.5 s	10.6 s [7 s–1 min 2 s]
Stellant single + multi-use	Total meantime for the injector preparation and the RIS and traceability = 109.65 s							
Centargo + SmartProtocol	47	37.3 s	37 s	7.38 s [22–62 s]	NA	NA	NA	NA

Differences in mean for injector preparation and RIS and traceability between Stellant single + imulti-use vs. Centargo + Smart Protocols + RIS connectivity = 72.35 s (on average, calculation: 109.65 − 37.30)

While Stellant (single-use and multi-use) injector preparation time is composed of 1 measurement, Centargo injector preparation time is composed of 2 measurements, the injector preparation and the protocol preparation while using Smart Protocols including weight-based and kV integration. Imputation of missing values for Centargo was done by replacing missing values with the corresponding mean values of the non-missing cases. The statistical calculation used a two-sample *t*-test for calculating the *p*-value from two-sided.

The difference between the Stellant systems and Centargo including injector preparation and ICM documentation for traceability activities is 72.35 s per patient (on average, calculation: 109.65 per patient − 37.30 per patient). If we place this difference in the context of a 10‐min (600-s) time period, which is the Theoretical time allocated of 10 min per patient/examination at CIMROD in CT Scanner, approximately 12% of that time is saved on average. If we extrapolate the time saved per patient to the mean number of injected exams per day per injector within CIMROD, 28, radiographers are saving about 35 min of their time per day.

The second and third secondary endpoints are closely related to ALARA principles. Specifically, the injector’s location is near the radiation source, which is the CT scanner. Although the radiographer only approaches the equipment when it is not emitting radiation, it is advisable to maintain a safe distance as a precaution. These automated systems help reduce radiographer to potential exposure to radiation by minimizing the time spent near the radioactive source (Table 4).

**Table 4:**
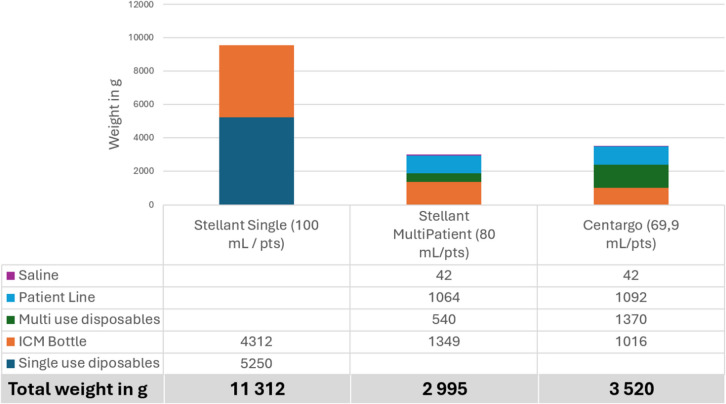
Theoretical measurement of waste production in gram according to the injector used based on an average of 28 patients injected per day on 2 injectors

Individual ICM bottle and saline bag weight in gram after use: 500 mL bottles = 254 g, 200 mL bottles = 179 g, 100 mL bottles = 154 g, and 1 L physiological bag = 21 g. Consumables’ individual weight in gram after use: Stellant day set multi-kit = 270 g, patient line Stellant = 38 g, Centargo day set = 685 g, patient line Centargo = 39 g, and Stellant single-use kit = 250 g. Weight was done during the 3 onsite measurements (April 25 and 26 for Stellant single use, May 6 and 7 for the Stellant multi-use, and July 10 and 11 for Centargo).

According to a theoretical number of 28 injected procedures per day and an optimization of ICM consumption between Stellant multi-use compared to Centargo, the daily production of waste weight evolves from 11,312 g with Stellant single use, so 404 g per patient, to 2995 g with Stellant multi-use, so 107 g per patient, to finally land to 3520 g with Centargo, so 126 g per patient. Centargo reduced by 69% waste weight production compared to Stellant Single Use and Stellant multi-use reduced by 74% waste weight production compared to Stellant Single Use. The higher waste weight production with Centargo compared with Stellant multi-use (2995 g with Stellant multi-use vs. 3520 g with Centargo) is mainly explained by the weight of the multi-use kit.

On an annual basis, compared to single-use Stellant injectors, Centargo and Stellant Multi-use systems enable a reduction of approximately 2.2 tons of waste, based on 28 injections per day and 7500 injections per year over 268 working days. While this may seem minor compared to the 700,000 tons of waste generated annually by hospitals [10], it significantly contributes to sustainability efforts.

An unpublished evaluation by a French expert body in medical waste management indicates that, in 2025, the cost of managing non-infectious medical waste is approximately €300 per ton [11]. In our study, the savings achieved through the optimization of ICM, the reduction in the number of consumables, and the decreased time required for waste collection by authorized personnel are estimated to be around €660 per year.

## Discussion

Given the increasing demands placed on radiology within the healthcare setting, optimizing workflow efficiencies and imaging procedures, in alignment with ALARA principles, can greatly benefit both patients and staff. The recent shift in the supply model and multi-use label of ICM in France [1] presents an opportunity to explore innovative approaches and technologies in CT suites, aimed at enhancing these areas. Contrast-enhanced CT remains a cornerstone of the diagnostic pathway, and with patient numbers rising each year, it is essential to investigate new methods for reducing CME and radiation exposure without compromising diagnostic image quality. Furthermore, radiographers’ time is a valuable resource within the radiology unit, and saving time in alignment with ALARA principles is highly advantageous.

Centargo demonstrates benefits in this study while maintaining expected image quality after interventions conducted by application specialists meanwhile reducing the volume of contrast media injected. This reduction minimizes the impact on patients’ renal function and enhances their safety. Centargo enables radiographers to dedicate more time to patients’ care. By relieving radiographers of non-value-added tasks compared to Stellant, they would concentrate more on patient interactions, fostering a patient-centered approach to care. This shift is particularly important in radiology, where patient anxiety and discomfort can significantly influence the overall experience and outcome. Unfortunately, no data on patient satisfaction were collected during this experimentation.

The radiology department also experiences clear cost benefits associated with ICM consumption optimization, potentially reducing electricity consumption (though not measured in this study), and minimizing waste production and its associated management costs (not measured either). These financial advantages contribute significantly to overall efficiency.

Sustainability benefits are reflected in a commitment to responsible resource utilization, including iodine, energy, and plastics, facilitated by multi-use strategies that reduce both unused and overused iodine. The reduction of waste production is increasingly critical in today’s healthcare environment to achieve sustainable practices and clearly associated with cost benefits.

The time saved for radiographers through automation, along with the unmeasured improvements in their quality of life and sense of purpose, which are most probably improved. Indeed, non-value-added tasks are minimized and time is redirected to value-added activities. In practice and as collected during discussions between the independent observer and radiographers, CIMROD’s seniors Radiographers are able to dedicate more time to educational activities with junior staff, they are also able to integrate intensive activity sessions, such as emergency or not planned exams, during 1- or 2-h slots without being too late compare to the initial planning. Additionally, this approach reduces the likelihood of having patients to be scanned at the theoretical end of their shifts.

While the findings are promising, it is essential to acknowledge the limitations of this study. The single-center design may restrict the applicability and generalizability of the results to other settings. Although there are no conscious variations in staffing, patient demographics, and institutional protocols in daily practices, the lack of control over these variables could introduce bias that influences outcomes. Additionally, the absence of data on Stellant multi-use ICM consumption represents a notable weakness.

Additionally, despite the widespread use of the technology, there may be potential errors associated with Ubudu’s tagging system for tracking the distribution of activity. Indeed, as described in the results, the daily average position is not normally distributed, and the number of average positions per day is different between the first and the second periods, finally, the unequal collection periods (12 vs. 72 days) raise reliability concerns.

Moreover, manually generated data regarding time spent on injector preparation, ICM documentation for traceability, and waste weight may lack accuracy, potentially impacting the overall reliability of the findings. The use of an unpublished source for the economic claims related to waste cost, the variability between private and public, site size and regional variation of waste price are another limitation.

To enhance external validity, future research should aim to replicate this study across multiple centers with better control of variables, validating the findings and assessing the broader applicability of Centargo injectors in diverse clinical environments.

## Conclusion

In conclusion, the transition to advanced injector technologies like Centargo, equipped with Smart Protocols and other innovative features, marks a significant advancement in the field of medical imaging and addresses some key associated challenges. As healthcare continues to evolve, decision-makers are increasingly interested in embracing innovations that enhance best practices through improved efficiency and cost-effectiveness, reduce environmental impact, and allow for more time to be potentially dedicated to patient care.

## Abbreviations

*ICM*: Iodinated contrast media
*ALARA*: As low as reasonably achievable
*kV*: Kilovolt
*RIS*: Radiology Information System
*CIMROD*: Centre d’Imagerie Médicale de la Dordogne
*CIRTACI*: Comité d’Information et de Recherche sur les Techniques d’Analyse Clinique
*CT*: Computed tomography
*OEM*: Original Equipment Manufacturer
*GDPR*: General Data Protection Regulation
